# The effects of affect: Working memory, rumination and emotion reactivity - Insights from an affective n-back task

**DOI:** 10.1371/journal.pone.0360749

**Published:** 2026-10-23

**Authors:** Nanna Karen Gilberg Skram, Constance Gaard Storvestre, Eivind Haga Ronold, Sheri L. Johnson, Matthew V. Elliott, Kenneth J.D. Allen, Åsa Hammar

**Affiliations:** 1 Department of Medical and Biological Psychology, University of Bergen, Bergen, Norway; 2 Department of Psychology, University of California, Berkeley, California, United States of America; 3 Department of Clinical Sciences Lund, Psychiatry, Faculty of Medicine Lund University, Lund, Sweden; 4 Department of Psychiatry, Skåne University Hospital, Lund, Sweden; University of Pennsylvania Perelman School of Medicine, UNITED STATES OF AMERICA

## Abstract

**Objective:**

Internalizing disorders are associated with emotion regulation characterized by repetitive negative thinking. There is a proposed relationship between rumination and working memory and there are gaps in understanding how emotions impact this association. In the present study, we investigated differences in negative (hot) and neutral (cold) working memory processing in relation to measures of rumination and emotion reactivity. Our aim was to address hot working memory as a potential clinically relevant mechanism in challenges with emotion regulation. Emotion reactivity was hypothesized to mediate the relationship between rumination and working memory.

**Method:**

Participants were undergraduate students (*N* = 120). Working memory was assessed using the Memory Affective Flexibility Task (MAFT), an affective experimental paradigm based on the *n*-back task that incorporates neutral and valenced visual stimuli. Facets of maladaptive emotion regulation were measured through self-report questionnaires for rumination and emotion reactivity. Associations were examined using multiple linear regression with rumination as the outcome variable.

**Results:**

There were small relationships between working memory performance in both neutral and negative conditions of the MAFT with measures of rumination and emotion reactivity. There was a significant moderate correlation between emotion reactivity and rumination.

**Conclusion:**

Despite correlations among self-report measures of emotion reactivity and rumination, we observed limited support for the links of task-based hot or cold working memory performance with self-rated emotion measures. This aligns with previous reports of modest correlations between task-based and self-report measures. The findings add to a growing literature on the challenges of capturing the interplay between emotion processes, affect and cognition in neuroscientific research.

## 1. Introduction

Internalizing disorders, such as depression and anxiety, have been shown to be robustly related to three key emotion processes: emotion reactivity, emotion regulation, and rumination [1–8], which in turn have each been shown to be crucially related to well-being and functional outcomes [9]. Whereas emotion reactivity is defined as a propensity to generate heightened responses to emotionally salient stimuli [10–14], emotion regulation is defined as processes that influence the magnitude, duration and expression of an emotional response [15]. Emotion processes are dependent on affective states, defined as pleasant or unpleasant feelings of valence and a level of arousal, which is considered continuously involved in cognition, including problem-solving and decision-making [16]. These processes are hypothesized to be associated with the cognitive deficits evident in internalizing disorders [17,18]. Rumination is a particularly relevant form of maladaptive emotion regulation, defined as passive repetitive negative thinking about past events without engagement to change one’s situation [3]. Research shows that trait-like tendencies to ruminate predict several clinical outcomes, including onset and maintenance of depression [3,19], depressive recurrence [20], and suicide [21]. It is therefore critical to gain an understanding of why some individuals ruminate, whereas others can disengage from their negative thoughts. Thus, the focus of this article is on enhancing the mechanistic understanding of rumination and emotion processes through investigation of its association with cognitive functioning.

Towards this aim, we focus on the role of working memory (WM). WM is a part of the separable but related cognitive processes of inhibition, shifting and updating/WM [22,23] that when grouped together, are coined executive functions (EF). EFs are well-validated as important for emotion regulation [18–25] and rumination [26–31]. WM in particular can be defined as a set of mental processes that make information temporarily available for processing, while inhibiting attention to distracting information [23,32]. Problem-solving, decision-making, and goal directed cognition tasks all rely on WM [33], and WM is therefore considered to be associated with the capacity of cognitive flexibility [34–36]. WM difficulties are particularly prominent in emotional contexts [20,24,29,32,37–39] and negative emotions can reduce WM capacity [13,14,24,40]. Results of a meta-analysis indicated that difficulties with both hot WM, (processing of affective stimuli), and cold WM, (processing of neutral stimuli), are both transdiagnostically related to psychopathology [14]. Several different cognitive tests have been used to assess hot and cold WM capacity [13,40] but the most frequently used task is the *n*-back [14]. The *n-*back paradigm is considered ideal for measuring the ability to discard information from WM [41]. Most research using the *n*-back paradigm finds that negative affective stimuli hinder WM capacity [12,14,42–45], an effect that is supported by neuroimaging studies [14,43,46], albeit one study indicated a performance decrement only when the negative emotion stimuli was irrelevant to the task [47]. In contrast, positive affective stimuli or task-relevant stimuli has been shown to improve WM capacity. Furthermore, positive stimuli used in laboratory studies of WM are considered low in affective significance and are found to be less reliable than unpleasant stimuli [14].

Rumination has been shown to be more specifically tied to WM difficulties than general EF impairment is [36], and WM deficits have been suggested as causative for rumination [35]. However, empirical findings on the role of hot WM for rumination are scarce, and findings on the effects of hot and cold WM with the *n*-back task in relation to trait rumination have been mixed [8,12,48]. More research is needed to investigate if impairments in updating hot WM may contribute to the onset and maintenance of rumination as it hinders effective emotion regulation [49,50], leading to sustained negative affect [19]. The ability to regulate negative mood states may distinguish individuals who spiral into a vicious cycle of increased negative thinking, which in turn deepens the negative mood state [29].

A significant relationship between emotion reactivity and trait rumination has not been consistently observed [2,51,52]. Although state and trait rumination are moderately correlated, only state rumination has been shown to be correlated to emotion reactivity and regulation [41,53]. High WM updating ability is found to be associated with less subjective arousal in response to negative affective stimuli [54]. Given that differences between hot and cold WM have been found across various paradigms, we suggest that trait emotion reactivity may be an associated construct and focus for further investigation. Bø et al. [51] further emphasize the importance of investigating emotion reactivity in relation to rumination and WM. In sum, emotion reactivity and rumination are key correlates of internalizing symptoms, and are both related to WM. While it is likely that WM can facilitate emotion regulation, at the same time, WM is particularly vulnerable to the effects of emotional valanced stimuli [37]. Taken together, bidirectional relationships of emotion reactivity, emotion regulation, and WM are expected. Although theory has emphasized the importance of hot WM difficulties, relatively little research has conjointly examined hot versus cold WM in relation to these clinically-relevant psychological constructs.

The aim of the present study is to investigate how hot and cold WM relate to rumination and emotion reactivity. High emotion reactivity could increase the challenge of disengaging from salient negative content in WM, which could provide a mechanistic explanation of trait rumination [6,28]. Thus, trait emotion reactivity, the tendency to be emotionally reactive, is a potential mediating factor between hot WM and trait rumination. The current analysis is particularly relevant given the hypothesized role of emotion reactivity, an often overlooked variable in this context, in explaining why some individuals’ WM is more affectively driven, “hot”, and consequently more susceptible to rumination. By exploring hot WM’s correlations with rumination and emotion reactivity, we aim to provide insights into individual differences in emotional regulation. Consequently, our focus is also on distinguishing between hot cognition in the setting of salient emotional contexts, and cold cognition, as purely cognitive [55,56]. We hypothesize that emotion reactivity and rumination correlate more strongly with hot (negative) than cold (neutral) WM. We also hypothesize that individuals with elevated emotion reactivity report higher levels of rumination.

## 2. Design, materials and methods

The current data was collected as part of a cross-sectional study. All study procedures were approved by the Institutional Review Board (IRB) of University of California Berkeley before data collection commenced. Ethical approval for the study was obtained from the Committee for Protection of Human Subjects at the University of California, Berkeley (CPHS protocol ID 2024-01-17038 & 2020-07-13459).The studies were conducted in accordance with the local legislation and institutional requirements. The participants provided their written informed consent to participate in this study. The hypotheses and analysis plan were preregistered before accessing the data (https://aspredicted.org/z7ch-b5kv.pdf). A previous publication describes initial psychometric analyses of the Memory and Flexibility Task (MAFT) in this sample [57] but does not consider emotion reactivity or rumination.

### 2.1 Participants and procedures

The study sample consisted of 120 undergraduate students, aged 18–47 years, recruited from a large public university and compensated with partial course credit for their participation. Interested individuals were recruited from a departmental research participant pool. Although all students in the pool were eligible to take part, one goal was to ensure an adequate number of participants with difficulties with Emotion-related impulsivity (ERI) (i.e., characterized by impulsive cognition and behavior in response to heightened emotions [58], captured by Negative and Positive Urgency scales. To ensure an adequate range of Urgency scores, students in the pool who elected to complete an online prescreening survey and scored greater than 3.5 out of 5 on the Urgency subscale of the abbreviated Urgency–Perseverance–Premeditation–Sensation-seeking–Positive urgency scale (UPPS-P) [59], were actively invited to participate. Measures were administered online, and they included 3 embedded items to capture poor attention (e.g., for data quality, please choose “1” for this item). After completing an online assessment of self-report measures, individuals who passed [at least 2 out of the 3 of the} attention check items were invited into the laboratory for a 2.5-hour session where they completed written informed consent procedures, and an assessment battery consisting of several computerized neuropsychological tasks, including the MAFT.

### 2.2 Measures

#### 2.2.1 *Emotion reactivity scale (ERS).*

Participants completed the ERS, a 21-item scale. Each ERS item is rated on a Likert-type scale ranging from 0 (“not at all like me”) to 4 (“completely like me”). The ERS has strong psychometric properties, including evidence of validity in the form of high correlations with psychopathology measures [60] and strong internal consistency in the current sample (α = .93).

#### 2.2.2 *Rumination response scale brooding subscale (RRS-B).*

Rumination was assessed using the 5-item ‘Brooding’ subscale of the RRS, which is considered a measure of maladaptive rumination unconfounded by depression content [61]. The Brooding subscale reflects passive comparisons to unattained standards and past-oriented patterns of repetitive negative thinking. Each item is rated (score range: 5–20) on a 4-point scale, ranging from 1 (“almost never”) to 4 (“almost always”), with higher scores indicating more ruminative tendencies. Previous studies using this measure have demonstrated strong test-retest reliability, as well as acceptable convergent and predictive validity [61–63].

#### 2.2.3 *Memory affective flexibility task.*

We measured WM ability using the MAFT [57]. The MAFT (Fig 1) is a timed, computer-based behavioral assessment that, to our knowledge, is the first n-back designed to probe WM and cognitive flexibility concurrently using affective and neutral stimuli, – see Allen and colleagues (2025) for full description [57]. The MAFT contained two main trial types: “n-back” trials and “switch” trials. “N-back” trials probed WM using a standard *n*-back procedure. That is, participants determined whether the current image was identical (“match”) or not (“mismatch”) to a target image displayed *n* trials earlier (range of *n =* 1–3). “Switch” trials probed cognitive flexibility. On “switch” trials, participants were instructed to rate the emotional valence (i.e., “positive” or “negative”) of the current image. Although the task was developed to assess WM and switching (cognitive flexibility), recent work casts doubt on the validity of the switching indices. Accordingly, we focus on n-back scores only here.

**Fig 1:**
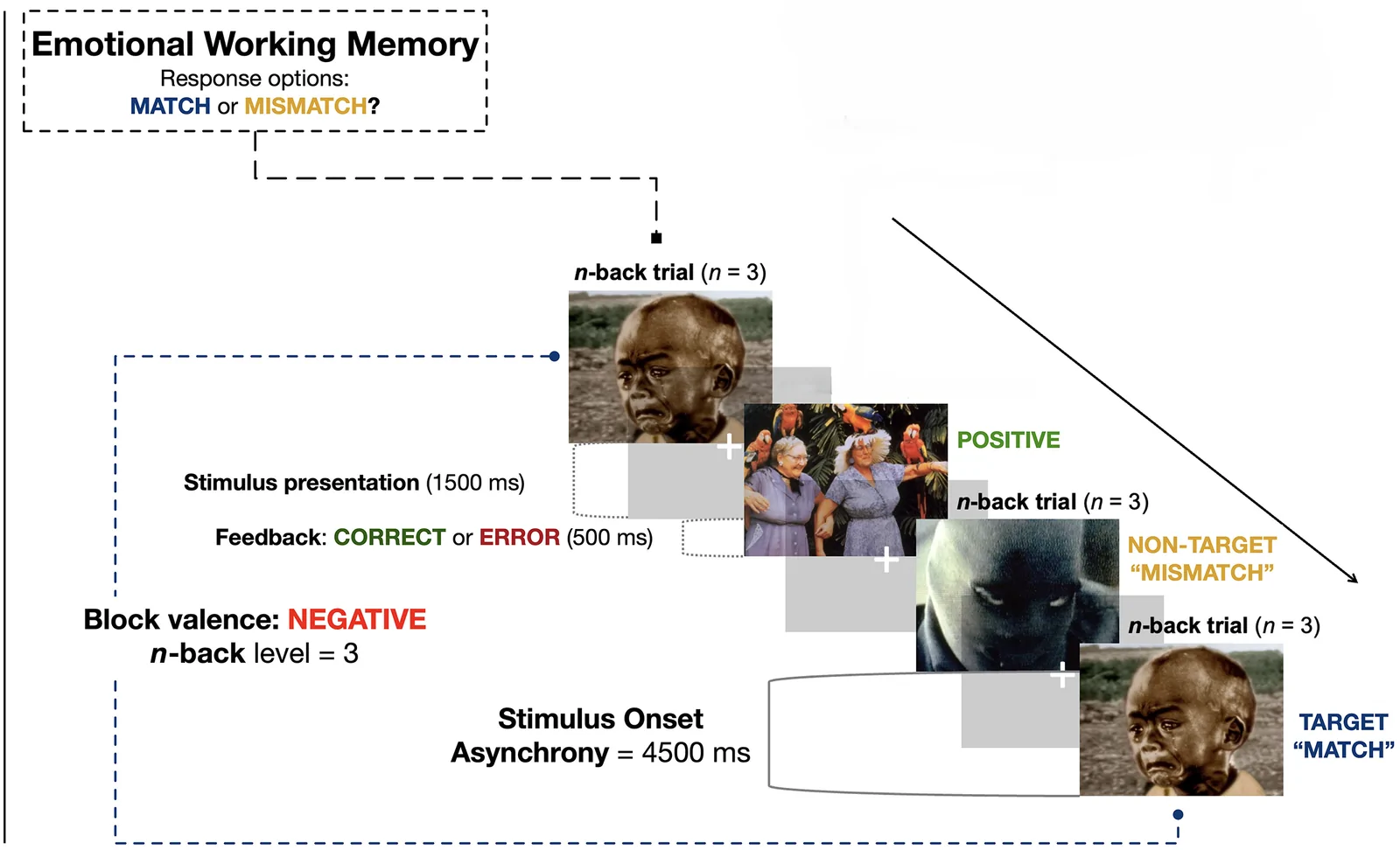
Memory and Affective Flexibility Task (MAFT). Note: Schematic overview of the *n*-back (*n* = 3) in the Memory and Affective Flexibility Task with affective and neutral images (MAFT; Adapted from Allen et al. [57]). Licensed under CC BY 4.0.

Before the experimental blocks, participants completed two practice blocks to learn the four-key response mapping – match, mismatch, positive and negative. On each trial, an image was presented on the screen for 1.5 seconds, and participants were given a 4.5 second response window. Trial-level feedback was presented for 500 ms during practice blocks only. Practice blocks were repeated until the participant achieved an accuracy threshold of at least 70% on WM trials to demonstrate comprehension at the *n* = 1 and *n* = 2 levels.

The MAFT included nine experimental blocks. Each block contained ~60% *n-*back (12 + n trials) and ~40% “switch” (8 trials). Blocks were divided in a 3 (*n*-level = 1, 2, or 3) by 3 (image valence = neutral, negative, or positive) design. The MAFT used standardized images from the IAPS [64]. Neutral images were chosen for their low arousal (M = 2.88, SD = 0.57) and intermediate valence (M = 4.98, SD = 0.30) on standardized ratings. The negative and positive image sets were selected for higher standardized ratings of arousal (negative: M = 5.94, SD = 0.77; positive: M = 5.22, SD = 1.02) and valence intensity (after reverse-coding for comparison; negative: M = 7.22, SD = 1.04; positive: M = 7.15, SD = 0.57) [56]. For each individual trial, an IAPS image was drawn randomly from the stimulus battery without replacement. The order of *n*-back and switch trials was pseudorandom to maintain the correct proportion in each block.

Given that the present hypotheses focus on the interplay of negative affect and WM capacity, we constrain our analyses to the WM trials with negative and neutral image stimuli. In addition, we do not include analyses of the MAFT “switch” trials in the present study as recent findings do not support this outcome noted above. The MAFT provides two summary metrics: (1) Accuracy, defined as the proportion of correct responses on the *n*-back trials (both “match” hits and “mismatch” rejections), and (2) Reaction Time (RT), which refers to the average speed at which correct responses are made across trials. Allen et al. [57] showed that WM performance was significantly reduced by negative stimuli compared to neutral stimuli on the MAFT and RT were significantly longer for the negative stimuli, indicating that the task was sensitive to the difference between hot and cold WM capacity for negative n-back trials compared to those with positive and neutral stimuli. In conclusion, WM performance showed reduced capacity under emotional stimuli exposure, aligning with prior research [12,14].

Allen et al. [57] found no evidence for a speed-accuracy tradeoff in bivariate correlations shown as negative correlations. No tradeoff is surprising as previous studies show that participants take extra time to respond correctly, while RT only measures time spent answering [47,65]. The limited associations suggest that RT may reflect individual differences in overall processing speed or response style rather than emotional valence-specific effects, as suggested in the preliminary validation of the MAFT (all r´s > 0.65). Allen et al. [57] demonstrated strong correlations among RT indices across all MAFT blocks and trial types, and suggested that RT might reflect speed of processing rather than functioning as a WM-specific index. Although accuracy was initially chosen as the most appropriate measure of WM performance in line with prior recommendations [47,57,65], we provide secondary tests of RT as the MAFT is a novel task.

### 2.3 Data analysis

Before conducting analyses of hypotheses, data were cleaned and checked for errors and statistical assumptions, in line with recommendations [66]. Data cleaning was performed using R version 4.4.1 [67], while statistical analyses and assumption checks were conducted in JASP, an open-source platform built on R scripts [68].

Bivariate correlations were provided for descriptive information before constructing multivariate models. As Shapiro Wilks test suggested non-normality Spearman’s correlations were used to examine the relationships among the key variables of ERS, RRS-B, and *n*-back accuracy and RT for negative and neutral stimuli. However, the Shapiro-Wilk test is sensitive to larger sample sizes [69]. Outliers for variables of accuracy and RT, (SD > +/-2.5) were adjusted using winsorization (i.e., replacing extreme values with the nearest value within the specified threshold to improve normality and reduce the influence of outliers [70] and reported in the Supporting Information (S4). There were no outliers for ERS and RRS-B. Residual plots and Q-Q plots indicated that residuals were approximately normally distributed with no severe deviations with the exception of the hot WM accuracy score. Homoscedasticity was confirmed through visual inspection of residual-versus-fitted plots. Multicollinearity was assessed using Variance Inflation Factors (VIF), with all values falling close to 1 (range: 1.002–1.335), indicating no multicollinearity concerns. Durbin-Watson statistics were within acceptable range across all models (range: 1.69–1.93), suggesting no problematic autocorrelation in the residuals. The assumptions of linear regression were considered sufficiently met to proceed with interpretation of the results. Regression coefficients (*β*) were interpreted as small.10, medium.30 and large.50 [71].

#### 2.3.1 *Deviations from the preregistration.*

The analyses were preregistered as Structural Equation Modelling (SEM) path models. However, following model estimation, fit indices indicated severe misspecification across all models (TIF <−4, CFI ≈ 0, RMSEA = 0.63, SRMR = 0.15), rendering the SEM results uninterpretable. When SEM models fail to converge to acceptable fit, regression has been recommended as a suitable and more interpretable alternative [72]. As a result, multiple linear regression was adopted as the primary analytical approach. The original SEM results are reported in Supporting Information (S5) for transparency. Two multiple linear regression models were conducted to examine whether n-back task performance predicted rumination above and beyond emotional reactivity. Model 1 included accuracy measures, Model 2 included reaction time measures.

ERS subscales were included with the intention to provide greater granularity in the analysis by distinguishing between different aspects of emotional reactivity, such as sensitivity, persistence, and arousal/intensity. However, the correlation matrix showed very strong overlap between the subscales and the overall ERS total score (ρs = .76–.95, all ps < .001), indicating that the measures were highly interrelated. See Table A1 in Supporting Information. As the 1-back condition places minimal demands on working memory and risks introducing ceiling effects that could obscure meaningful variation in performance, it was excluded from further analyses. The 2-back and 3-back conditions were therefore examined to assess whether increasing cognitive load in the 3-back condition added meaningful variance beyond 2-back. Wilcoxon signed-rank tests were conducted comparing performance across conditions. Statistically significant differences were found for neutral accuracy (*W* = 2124, z = 2.656, *p* = .005, *r* = .344), negative accuracy (*W* = 2101, *z* = 2.305, *p* = .016, *r* = .297), and neutral RT (*W* = 2128, *z* = −2.883, *p* = .004, *r* = −.315), while negative RT did not differ significantly (*W* = 2538, *z* = −1.091, *p* = .276, *r* = −.121). Despite these differences, effect sizes were small to medium in magnitude (*rs* = .121–.344), and the directional pattern of performance was consistent across conditions. See Table A2 in Supporting Information. Although some statistical differences were observed, the overall pattern of results was highly similar across conditions. See Table A3 in Supporting Information. Given this similarity, a composite score averaging performance across the hot and cold 2-back and 3-back conditions was computed for subsequent analyses.

## 3 Results

### 3.1 Preliminary analysis

All participants attained < 50% omission error rates on MAFT *n*-back trials. Final analyses were restricted to *n* = 120 participants. See Table 1 for demographic data. See Table 2 for descriptive statistics on key variables. Before analyses of hypotheses, distributional properties were examined using the Shapiro-Wilk test. Accuracy variables showed significant deviations from normality (all ps < .001). Log10 transformation did not resolve the non-normality of accuracy variables (all ps < .001 post-transformation), likely reflecting genuine ceiling effects. As linear regression assumes normally distributed residuals rather than predictors, and residual normality was confirmed through Q-Q plot inspection etc. described above, and untransformed variables were retained in all analyses.

**Table 1:** Demographic characteristics (N = 120).

	*Mean*	*SD*
**Age**	21.45	4.14
Education in Year	14.45	1.36
**Gender Identity**	*n*	%
Female	104	86.67%
Male	15	12.50%
Nonbinary	1	0.83%

*Note:* Race/Ethnicity: American Indian or Alaskan Native (1.67%), Asian (45%), Black or African (0.83%), Hispanic, Latino/a, or Spanish (10.83%), Middle Eastern or North African (2.50%), White (24.17%), Mixed race or Multiracial (13.33%), Not reported/other (1.67%).

**Table 2:** Descriptive statistics (N = 120).

	Mean	SD	Skewness	Kurtosis	Shapiro-Wilk	Minimum	Maximum
ERS	60.13	20.34	0.35	−0.57	W = 0.97, p = 0.011	24.00	105.0
RRS-B	12.93	3.82	−0.01	−0.72	W = 0.98, p = 0.041	5.00	20.00
MAFT ACC neutral	0.83	0.14	−1.021	1.2320	W = 0.86, p=<.001	0.25	1.00
MAFT ACC Negative	0.68	0.20	−0.53	−0.42	W = 0.94, p=<.001	0.00	1.00
MAFT RT neutral	1128	199.0	0.54	−0.04.26	W = 0.98, p = 0.03	706.3	1708
MAFT RT negative	1119	204.6	0.20	−0.192	W = 0.99, p = 0.760	584.8.5	1669

*Note.* All participants, including outliers. RRS-B = Rumination Response Scale Brooding subscale,

3 participants were missing RRS-B, 1 participant was missing ERS. ERS = Emotion Reactivity Scale, ACC = accuracy = % correct. RT = reaction time in ms.

As shown in Table 2, WM accuracy scores in the hot and cold conditions had limited ranges and showed overall high accuracy rates, indicating ceiling effects on WM performance.

As shown in Table 3, RT scores were highly correlated across MAFT valence conditions. MAFT accuracy for negative and neutral blocks was modestly correlated, as was MAFT RT. Emotion reactivity (ERS) and rumination (RRS-B) were significantly and moderately correlated. Neither ERS nor RRS-B showed significant bivariate correlations with any of the MAFT indices (See Table 3). Whether emotion reactivity mediated rumination and working memory was not tested due to the link of rumination and working memory was null (Table 4).

**Table 3:** Bivariate Spearman’s correlation coefficients (N = 120).

Variable		1	2	3	4	5	6
1. RRS-B	Spearman’s rho	—					
2.ERS	Spearman’s rho	0.555***	—				
3.MAFT ACC neutral	Spearman’s rho	−0.018	0.021	—			
4.MAFT ACC Negative	Spearman’s rho	−0.030	0.011	0.117	—		
5.MAFT RT neutral	Spearman’s rho	0.093	−0.017	−0.298**	−0.065	—	
6. MAFT RT negative	Spearman’s rho	−0.048	−0.054	−0.193*	−0.061	0.473***	—

*Note.* All participants, including outliers. ACC: Accuracy measured as percent correct, RT: – Mean reaction time in ms.

* *p* < .05, ** *p* < .01, *** *p* < .001

**Table 4:** Multiple linear regression.

	Model 1		Model 2	
	β	p	β	p
ERS	0.55	<.001	0.55	<.001
MAFT ACC neutral	−0.50	0.537	—	—
MAFT ACC Negative	−0.02	0.810	—	—
MAFT RT neutral	—	—	0.16	0.076
MAFT RT negative	—	—	−0.08	0.365
R²	0.31		0.32	
Adjusted R²	0.29		0.30	
F	15.86	<.001	16.08	<.001

*Note*. Standardised regression coefficients (*β*) are reported. ERS = Emotional Reactivity Scale; RT = reaction time; Acc = accuracy.

### 3.2 Regression models

#### 3.2.1 *Model 1: Accuracy.*

A multiple linear regression with emotion reactivity, accuracy on the N-back working memory neutral and negative trials as predictors of rumination was statistically significant, F(3, 106) = 15.86, p < .001, explaining 31.0% of the variance (R² = .310, Adjusted R² = .290, RMSE = 3.141). ERS was the only significant predictor, and it showed a large effect (β = .555, B = 0.105, SE = 0.015, t = 6.88, p < .001), indicating that higher emotional reactivity was associated with greater rumination. Neither neutral WM accuracy (β = −.050, B = −1.315, SE = 2.126, t = −0.62, p = .537) nor negative WM accuracy (β = −.020, B = −0.377, SE = 1.561, t = −0.24, p = .810) significantly predicted rumination when controlling for ERS.

#### 3.2.2 *Model 2: Reaction time.*

A parallel multiple linear regression with emotion reactivity (ERS), and neutral, and negative WM RT scores as predictors also was statistically significant, F(3, 101) = 16.08, p < .001, explaining 32.3% of the variance in rumination (R² = .323, Adjusted R^2^ = .303, RMSE = 3.069). Also parallel with the accuracy model, ERS was the only significant predictor (β = .550, B = 0.101, SE = 0.015, t = 6.71, p < .001). Neither neutral WM RT (β = .164, B = 0.003, SE = 0.002, t = 1.79, p = .076) nor negative WM RT scores were significant predictors (β = −.083, B = −0.001, SE = 0.002, t = −0.91, p = .365) when controlling for ERS.

#### 3.2.3 *Summary.*

Across regression models, emotional reactivity (ERS) consistently and significantly predicted rumination (RRS-B). N-back task performance variables did not contribute significant unique predictive variance beyond emotional reactivity in any model.

## 4. Discussion

In the present study, we investigated the relationship between behavioral measures of hot and cold WM and self-reported rumination and emotion reactivity. Contrary to predictions, we did not observe significant associations of either hot or cold WM indices with rumination or emotion reactivity.

Considering theory and previous findings linking WM capacity with emotion regulation [36], and rumination [14,35,36,73], the present results are somewhat surprising. If the absence of an association represents true nulls, questions related to the interplay between generated emotional reactions, regulated emotions and cognitive functioning can be raised. The distinction between emotion reactivity and emotion regulation on a neurological level is therefore a point of interest. On a broader theoretical, biological and psychological level [74, 75], the distinction between emotion reactivity and emotion regulation have been assumed to involve separable brain systems where emotion reactivity stems from subcortical regions and regulation occurs in higher frontoparietal areas of the cortex [76, 77], referred to as the neural separability hypothesis [76,78]. In this study emotion reactivity does not appear to be associated with the higher cortical function of WM. Interestingly, recent empirical investigations of the neural separability hypothesis do not find consistent directions between brain areas involved in emotion reactivity vs regulation [76].

Beyond considering the role of hot WM, our second hypothesis was that individuals who endorsed higher levels of rumination would report elevated emotion reactivity, and this hypothesis was supported by the moderate to large significant association we observed. This association is not surprising, as rumination is considered a maladaptive emotion regulation strategy which is recruited in moments of high emotion. Our findings are parallel with those of some previous studies [79] including experimental research which suggests that inducing intense emotion can provoke rumination [80]. Both processes are related to psychopathology and emotion regulation [51]. Although our cross-sectional design does not support statements on the directionality of the relationship, our finding is consistent with theory suggesting that reactivity or distress can trigger rumination as a maladaptive attempt to understand and regulate emotions [3,62]. In light of the neural separability hypothesis and distinguishing emotion reactivity and maladaptive regulation in the form of rumination, this study does not find an association to WM.

One relatively novel focus for the current study was the use of a hot WM task. Here, then, we focus on the unexpected lack of association between rumination and hot WM, as this is a previously established association [35,36]. Since bivariate correlations were small, the null results do not appear to be explained merely by statistical power. The correlations and the regressions provided no indications that hot WM was a more powerful correlate of rumination than cold WM is. Indeed, we saw no significant links between hot WM with any of our self-rated emotion variables. Null results always raise questions about reliability, but MAFT scores have shown modest consistency in individual performance across blocks [57] and expected decays in performance and speed with the negative trials as compared to the positive trials. One possibility is that the nature of our hot WM task limited effects, in that studies relying on the affective n-back studies have varied in whether they found significant correlations of hot WM with trait rumination [8,14,49].

Our results could reflect the broader conceptual and empirical problem of a disconnect between self-report measures and controlled behavioral tasks. That is, it is unclear if the null correlation reflects methodological limitations or a true lack of association [81]. Our findings are in this regard consistent with previous literature [82,83]. Self-report measures are considered generally reliable, but response styles can influence outcomes, whereas behavioral tasks often show greater variability and lower reliability [84,85]. The disconnect between subjective and objective measures is potentially linked to task-impurity which is well-documented in EF tasks [22,83], which may be relatively nonspecific as they are found to tap into multiple EF and non–EF processes [81]. Although this can be addressed using latent models of multiple EF tasks [86]. Even latent behavioral variables often show small associations with self-report measures [81,87]. Divergence between self-report and behavioral measures may also reflect that self-report measures tend to capture typical performance across a range of contexts (i.e., trait), whereas behavioral tasks more typically capture performance in optimal and controlled conditions (i.e., state) [88]. Nonetheless, introducing affective stimuli in a behavioral task also comes with its own set of challenges both conceptually and methodologically [36].

While it is possible that hot WM is not important for rumination or emotion reactivity, our profile of null results for the MAFT raises methodological and validity questions for this relatively novel task. Accuracy was initially chosen as the most appropriate measure for WM capacity. Nonetheless, ceiling effects in accuracy scores andlimited variability likely reflected that more difficult trials should have been conducted. Given this, we conducted sensitivity analyses using RT as the measure for WM capacity. The development of more precise and reliable psychometric measures of EF are frequently emphasized in the literature [22,24,89]. The MAFT is one such attempt, aiming to capture individual differences in WM capacity in affective and non-affective contexts. However, our findings do not reveal if the MAFT targets the core of hot cognition and whether the salience of affective stimuli elicit strong affective responses. Arousal invoking images are frequently presented in today’s media, such that participants could be habituated with similar stimuli. The use of standardized stimuli may also diminish the autobiographical relevance, thereby diminishing affective salience [14,90]. The subjective experience of an affective response varies between individuals and is often difficult to capture and quantify [91]. To ensure that an affective response was present, our study design could have benefitted from incorporating psychophysiological measures (e.g., skin conductance or heart rate) and subjective self-report questionnaires [40], or from using tasks that invoke stress, such as personal failure feedback or personally relevant affective stimuli [14,40,41,51,92].

### 4.1 Strengths and limitations

The current study is distinguished by the multivariate model of emotion-related processes, and the use of an innovative experimental paradigm to examine the influence of affect on WM [51,57]. Our findings support the hypothesized link between trait rumination and emotion reactivity. However, study limitations suggest that the results should be interpreted with caution. The cross-sectional design of the study limits conclusions about the direction of effects, and the results indicate that the MAFT did not challenge the participants sufficiently.

There are implications of a nonclinical, largely female, sample that was selected without regard to diagnostic status that should be addressed. Even though the participants were over-sampled for emotion regulation difficulties, few participants endorsed extreme difficulties with emotion reactivity or rumination and the homogeneity in emotion regulation may have artifactually constrained our ability to detect effects. However, rumination scores in the current population were higher than what is found in healthy controls [93]. Furthermore, college students have been found to have rates of depression and anxiety comparable to the general population [94]. Taken together with the diverse ethnicities present in this sample, we consider this sample representative of common emotion regulation difficulties.

Although this sample yielded significant differences between hot and cold WM [57], our null results may relate to the limited range of affect and cognitive difficulties within our nonclinical sample. Indeed, in recent meta-analyses, small correlations of measures of emotion regulation and (cold) EF have been attributed to the relative lack of variability in nonclinical samples [36,95]. Previous research has also demonstrated that affective information processing disrupts WM performance more profoundly in clinical samples [14,20,28,39,96]. Disruptions of WM and heightened vulnerability to affectively salient information align with broader deficits in WM observed across clinical groups [25,97]. Aside from the methodological limitations of our study, the limited number of measures restricted our ability to explore alternative models. As one example, other facets of executive control, such as response inhibition, have been suggested to be of importance in understanding emotion regulation [98].

### 4.2 Conclusions and future directions

The study found a moderate to large association between rumination and emotion reactivity. However, our model of the relation of hot WM with rumination and emotion reactivity was not supported. This might be explained by methodological challenges, such as the homogeneity in the sample or the experimental task we used to assess hot WM. Real time assessments of emotion regulation in ecological settings, in clinical samples, might better capture the underlying mechanisms of emotional regulation and the relationship to hot WM. Improving the understanding of hot WM can contribute to advancements in cognitive remediation training, highlighting its clinical potential. Furthermore, knowledge of the effects of affect on WM can inform clinicians of their patients’ current processing abilities and help patients understand how affect impacts their everyday life functioning.

## Supporting information

S1 Appendix Supplementary Analyses. (DOCX)

S1 File Inclusivity-in-global-research- Effect of Affect. (DOCX)
