# Supplementary material for: The effects of affect: Working memory, rumination and emotion reactivity - Insights from an affective n-back task
Source: PLoS One. 2026 Oct 23;21(10):e0360749. doi: 10.1371/journal.pone.0360749 (PMC13693938; doi:10.1371/journal.pone.0360749)
Supplement: Supplementary file 1 [file pone.0360749.s001.docx]

**Supporting Information**

S1 Appendix. Supplementary Analyses

**Table A1 Descriptives ERS subscores**

|  | Mean | SD | Skewness | Kurtosis | Shapiro-Wilk | Minimum | Maximum |
| --- | --- | --- | --- | --- | --- | --- | --- |
| ERS persistence | 3.14 | 1.07 | 0.18 | -1.06 | W=0.96,  p=0.002 | 1.00 | 5.00 |
| ERS arousal | 2.91 | 1.07 | 0.25 | -0.76 | W=0.97,  p=0.006 | 1.00 | 5.00 |
| ERS  sensitivity | 2.76 | 0.99 | 0.36 | -0.36 | W=0.96,  p=0.028 | 0.90 | 5.00 |

*Note.* All participants, including outliers. 2 participant was missing Persistence, 2 participant was missing Arousal, 2 participant was missing Sensitivity, ERS = Emotion Reactivity Scale.

**Table A2**

*Descriptives n2 and n3 separated*

|  | Missing | Mean | SD | Skewness | Kurtosis | Shapiro-Wilk | Minimum | Maximum |
| --- | --- | --- | --- | --- | --- | --- | --- | --- |
| n2 MAFT ACC neutral | 9 | 0.87 | 0.19 | -1.13 | -0.23 | W=0.71, p<.001 | 0.25 | 1.00 |
| n3 MAFT ACC neutral | 9 | 0.79 | 0.22 | -0.92 | -0.55 | W=0.81,  p<.001 | 0.00 | 1.00 |
| n2 MAFT ACC negative | 9 | 0.71 | 0.23 | -0.63 | -0.04 | W=0.86,  p<.001 | 0.00 | 1.00 |
| n3 MAFT ACC negative | 9 | 0.64 | 0.27 | -0.23 | -0.76 | W=0.89,  p<.001 | 0.00 | 1.00 |
| n2 MAFT RT neutral | 9 | 1091 | 2.45.3 | 0.88 | -1.894 | W=0.96,  p=0.160 | 615.0 | 2074 |
| n3 MAFT RT neutral | 10 | 1169 | 262.2 | 1.35 | 4.97 | W=0.92,  p=0.040 | 611.8 | 2491 |
| n2 MAFT RT negative | 11 | 1101 | 259.2 | 0.47 | 0.24 | W=0.98,  p=0.118 | 505.3 | 1856 |
| n3 MAFT RT negative | 12 | 1140 | 236.5 | -0.04 | -0.20 | W=0.99,  p=0.72 | 529.7 | 1863 |

*Note.* All participants, including outliers, ACC = accuracy = % correct. RT = reaction time in ms.

**Outliers (S4)**

Outliers for accuracy and RT variables (SD > ±2.5) were identified and adjusted using winsorization, replacing extreme values with the nearest value within the specified threshold to improve normality and reduce the influence of outliers [71]. In total, winsorization was applied to 10 cases, affecting accuracy variables (n2 neutral: *n* = 1; n3 neutral: *n* = 1; n2 negative: *n* = 1; n3 negative: *n* =1) and RT variables (n2 neutral: *n* = 2; n3 neutral: *n* = 1; n2 negative: *n* = 1; n3 negative: *n* = 2).

**Table A3 Wilcoxon signed-rank tests comparing 2-back and 3-back performance.**

Pairwise comparisons of accuracy and RT between 2-back and 3-back conditions across cold and hot stimulus valences. Effect sizes are reported as rank-biserial correlations (*r*). Normality of difference scores was assessed using the Shapiro-Wilk test prior to analysis.

| **Comparison** | ***W*** | ***z*** | ***p*** | ***r*** |
| --- | --- | --- | --- | --- |
| MAFT ACC neutral | 2124 | 2.656 | .005 | .344 |
| MAFT ACC negative | 2101 | 2.305 | .016 | .297 |
| MAFT RT neutral | 2128 | −2.883 | .004 | −.315 |
| MAFT RT negative | 2538 | −1.091 | .276 | −.121 |

*Note.* Wilcoxon signed-rank test. *r* = rank-biserial correlation. The Shapiro-Wilk test indicated that difference scores for accuracy comparisons violated normality assumptions (MAFT ACC neutral: *W* = 0.926, *p* < .001; MAFT ACC negative: *W* = 0.935, *p* < .001), warranting the use of non-parametric tests. Difference scores for RT comparisons were approximately normally distributed (MAFT RT neutral: *W* = 0.983, *p* = .156; MAFT RT negative: *W* = 0.978, *p* = .068).

**Structural Equation Modelling Analyses (S5)**

The path model (see Figure 1) did not support accuracy as a measure of hot and cold WM performance related to ERS and RRS demonstrated poor fit to the data, χ²(1) = 28.673, *p* < 0.001; CFI = 0.00; TLI = - 4.755; RMSEA = 0.518 (90% CI [0.366, 0.690]); SRMR = 0.133, and large error terms. The path model with RT as a measure of WM performance with age outliers excluded (see Figure 3; See Appendix with age outliers included) to examine whether results remained stable, did not improve model fit, χ² (1)= 28.359, *p* < 0.001; CFI = 0.00; TLI = - 4.701; RMSEA = 0.526 (90% CI [0.370, 0.701]); SRMR = 0.134. Thus, there was no substantial differences between the two models in terms of fit (Δχ²= 0.314).

**Figure A1**

## *SEM path model of WM accuracy with rumination and emotion reactivity.*

*Note*. RRS-B= Rumination Response Scale Brooding, ERS = Emotion Reactivity Scale, Acc Negative= Accuracy for negative trials, Acc Neutral= Accuracy for neutral trials. Standardized regression coefficients (β) shown.

**Figure A2**

*SEM path model of WM RT with rumination and emotion reactivity*

*Note*. RRS-B = Rumination Response Scale-Brooding, ERS = Emotion Reactivity Scale, RT Negative= reaction time for negative trials, RT Neutral = reaction time for neutral trials. Standardized regression coefficients (β) shown.
