# Supplementary material for: The effects of affect: Working memory, rumination and emotion reactivity - Insights from an affective n-back task
Source: PLoS One. 2026 Oct 23;21(10):e0360749. doi: 10.1371/journal.pone.0360749 (PMC13693938; doi:10.1371/journal.pone.0360749)
Supplement: Peer review history

# Peer review: Decision Letter 0

**DOI:** 10.1371/journal.pone.0360749.r001

**Authors:** Desmond J. Oathes

22 Jan 2026

Dear Dr. Ronold,

Thank you for submitting your manuscript to PLOS ONE. After careful consideration, we feel that it has merit but does not fully meet PLOS ONE’s publication criteria as it currently stands. Therefore, we invite you to submit a revised version of the manuscript that addresses the points raised during the review process.

Please submit your revised manuscript by Mar 08 2026 11:59PM. If you will need more time than this to complete your revisions, please reply to this message or contact the journal office at plosone@plos.org. When you're ready to submit your revision, log on to https://www.editorialmanager.com/pone/ and select the 'Submissions Needing Revision' folder to locate your manuscript file.

- A letter that responds to each point raised by the academic editor and reviewer(s). You should upload this letter as a separate file labeled 'Response to Reviewers'.
- A marked-up copy of your manuscript that highlights changes made to the original version. You should upload this as a separate file labeled 'Revised Manuscript with Track Changes'.
- An unmarked version of your revised paper without tracked changes. You should upload this as a separate file labeled 'Manuscript'.

If you would like to make changes to your financial disclosure, please include your updated statement in your cover letter. Guidelines for resubmitting your figure files are available below the reviewer comments at the end of this letter.

If applicable, we recommend that you deposit your laboratory protocols in protocols.io to enhance the reproducibility of your results. Protocols.io assigns your protocol its own identifier (DOI) so that it can be cited independently in the future. For instructions see: https://journals.plos.org/plosone/s/submission-guidelines#loc-laboratory-protocols. Additionally, PLOS ONE offers an option for publishing peer-reviewed Lab Protocol articles, which describe protocols hosted on protocols.io. Read more information on sharing protocols at https://plos.org/protocols?utm_medium=editorial-email&utm_source=authorletters&utm_campaign=protocols.

We look forward to receiving your revised manuscript.

Kind regards,

Desmond J. Oathes

Academic Editor

PLOS One

Journal Requirements:

When submitting your revision, we need you to address these additional requirements.

1. Please ensure that your manuscript meets PLOS ONE's style requirements, including those for file naming. The PLOS ONE style templates can be found at

https://journals.plos.org/plosone/s/file?id=wjVg/PLOSOne_formatting_sample_main_body.pdf and

https://journals.plos.org/plosone/s/file?id=ba62/PLOSOne_formatting_sample_title_authors_affiliations.pdf

2. Please include a complete copy of PLOS’ questionnaire on inclusivity in global research in your revised manuscript. Our policy for research in this area aims to improve transparency in the reporting of research performed outside of researchers’ own country or community. The policy applies to researchers who have travelled to a different country to conduct research, research with Indigenous populations or their lands, and research on cultural artefacts. The questionnaire can also be requested at the journal’s discretion for any other submissions, even if these conditions are not met.  Please find more information on the policy and a link to download a blank copy of the questionnaire here: https://journals.plos.org/plosone/s/best-practices-in-research-reporting. Please upload a completed version of your questionnaire as Supporting Information when you resubmit your manuscript.

3. Thank you for stating the following in the Acknowledgments Section of your manuscript:

“This work was funded by a grant from the Peder Sather Foundation and the corresponding author submitted the manuscript while on a Fulbright scholarship to UC Berkeley. The University of Bergen funded the work through coverage of the article processing charges.”

We note that you have provided funding information that is not currently declared in your Funding Statement. However, funding information should not appear in the Acknowledgments section or other areas of your manuscript. We will only publish funding information present in the Funding Statement section of the online submission form.

Please remove any funding-related text from the manuscript and let us know how you would like to update your Funding Statement. Currently, your Funding Statement reads as follows:

“The Peder Saether Foundation provided support for participant payment and for two meetings of the authors. Corresponding author is a visiting researcher at UC Berkeley on a Fulbright scholarship at the time of submission. The University of Bergen funded the article processing charges.”

Please include your amended statements within your cover letter; we will change the online submission form on your behalf.

4. We note that the grant information you provided in the ‘Funding Information’ and ‘Financial Disclosure’ sections do not match.

When you resubmit, please ensure that you provide the correct grant numbers for the awards you received for your study in the ‘Funding Information’ section.

5. Thank you for stating the following financial disclosure:

“The Peder Saether Foundation provided support for participant payment and for two meetings of the authors. Corresponding author is a visiting researcher at UC Berkeley on a Fulbright scholarship at the time of submission. The University of Bergen funded the article processing charges.”

Please state what role the funders took in the study.  If the funders had no role, please state: "The funders had no role in study design, data collection and analysis, decision to publish, or preparation of the manuscript."

If this statement is not correct you must amend it as needed.

Please include this amended Role of Funder statement in your cover letter; we will change the online submission form on your behalf.

6. When completing the data availability statement of the submission form, you indicated that you will make your data available on acceptance. We strongly recommend all authors decide on a data sharing plan before acceptance, as the process can be lengthy and hold up publication timelines. Please note that, though access restrictions are acceptable now, your entire data will need to be made freely accessible if your manuscript is accepted for publication. This policy applies to all data except where public deposition would breach compliance with the protocol approved by your research ethics board. If you are unable to adhere to our open data policy, please kindly revise your statement to explain your reasoning and we will seek the editor's input on an exemption. Please be assured that, once you have provided your new statement, the assessment of your exemption will not hold up the peer review process.

7. Your ethics statement should only appear in the Methods section of your manuscript. If your ethics statement is written in any section besides the Methods, please move it to the Methods section and delete it from any other section. Please ensure that your ethics statement is included in your manuscript, as the ethics statement entered into the online submission form will not be published alongside your manuscript.

8. We note that Figure 1 in your submission contain copyrighted images. All PLOS content is published under the Creative Commons Attribution License (CC BY 4.0), which means that the manuscript, images, and Supporting Information files will be freely available online, and any third party is permitted to access, download, copy, distribute, and use these materials in any way, even commercially, with proper attribution. For more information, see our copyright guidelines: http://journals.plos.org/plosone/s/licenses-and-copyright.

We require you to either (1) present written permission from the copyright holder to publish these figures specifically under the CC BY 4.0 license, or (2) remove the figures from your submission:

1. You may seek permission from the original copyright holder of Figure(s) [#] to publish the content specifically under the CC BY 4.0 license.

We recommend that you contact the original copyright holder with the Content Permission Form (http://journals.plos.org/plosone/s/file?id=7c09/content-permission-form.pdf) and the following text:

“I request permission for the open-access journal PLOS ONE to publish XXX under the Creative Commons Attribution License (CCAL) CC BY 4.0 (http://creativecommons.org/licenses/by/4.0/). Please be aware that this license allows unrestricted use and distribution, even commercially, by third parties. Please reply and provide explicit written permission to publish XXX under a CC BY license and complete the attached form.”

Please upload the completed Content Permission Form or other proof of granted permissions as an "Other" file with your submission.

In the figure caption of the copyrighted figure, please include the following text: “Reprinted from [ref] under a CC BY license, with permission from [name of publisher], original copyright [original copyright year].”

2. If you are unable to obtain permission from the original copyright holder to publish these figures under the CC BY 4.0 license or if the copyright holder’s requirements are incompatible with the CC BY 4.0 license, please either i) remove the figure or ii) supply a replacement figure that complies with the CC BY 4.0 license. Please check copyright information on all replacement figures and update the figure caption with source information. If applicable, please specify in the figure caption text when a figure is similar but not identical to the original image and is therefore for illustrative purposes only.

9. If the reviewer comments include a recommendation to cite specific previously published works, please review and evaluate these publications to determine whether they are relevant and should be cited. There is no requirement to cite these works unless the editor has indicated otherwise.

[Note: HTML markup is below. Please do not edit.]

Reviewers' comments:

Reviewer's Responses to Questions

**Comments to the Author**

1. Is the manuscript technically sound, and do the data support the conclusions?

Reviewer #1: Yes

Reviewer #2: Partly

Reviewer #3: No

**********

2. Has the statistical analysis been performed appropriately and rigorously?

Reviewer #1: Yes

Reviewer #2: No

Reviewer #3: No

**********

3. Have the authors made all data underlying the findings in their manuscript fully available?

The PLOS Data policy

Reviewer #1: Yes

Reviewer #2: No

Reviewer #3: Yes

**********

4. Is the manuscript presented in an intelligible fashion and written in standard English?

Reviewer #1: Yes

Reviewer #2: Yes

Reviewer #3: Yes

**********

Reviewer #1: This study sought to assess the relationship between emotional working memory, trait emotional reactivity, and trait rumination. This is an important area of study with interest to a wider readership including both clinical and non-clinical psychologists. I commend the authors for preregistering this study and providing straightforward, transparent descriptions throughout. I will stress that I do not believe the outcome of the study (primarily null) should be considered as part of the editorial decision. Nonetheless, I still have major and minor concerns which I list below.

Major

While commendable for preregistering the study, I cannot seem to access the preregistration document. A publicly-accessible link is necessary in order to critically evaluate adherence to preregistered analyses.

It’s not entirely clear to me why an SEM approach was adopted. I think the justification could be made a lot clearer. Additional, non-preregistered exploration of relationships within a regression or partial spearman rank framework could be insightful. Moreover, was age or any other demographic variables included as covariates?

Many of the measures and analyses might benefit from more granularity. The stimuli, subjective measures, and behavioral measures are quite broadly categorized and averaged. For example, the stimuli have been split into negative vs neutral stimuli. However, the impact of threat-relevant stimuli (inducing fear/anxiety) will likely have very different impacts on cognition to sad or disgusting stimuli. Summary statistics were also used for questionnaires and n-back data. Might other ways of processing such data give more detailed insight? For example, d prime or drift diffusion modeling of n-back data? Focusing on questionnaire items which showed relatively larger variability and less floor effects? I am not suggesting conducting a myriad of tests or any form of data fishing. Rather, considering a select number of supplementary, non pre-registered analyses that the researchers think could be useful. Such analyses could either: 1) reveal preliminary evidence for more nuanced relationships otherwise obscured by more “simple” analyses, with any inferences described as tentative; or 2) support the notion that these represent true null effects, no matter which way the data is analyzed.

The null results are largely discussed in the context of potential methodological limitations. This is important to keep in the discussion. However, it would be particularly beneficial if there was a paragraph or two commenting on what these results mean at face value. In other words, if these results represent a true null, what does this tell us about our theories of emotion and working memory? It is equally important to consider the possibility of null results being either true nulls or false negatives.

Minor

It might be helpful to briefly introduce hot vs cold memory in the abstract.

Be clear on the distinction between general executive functioning and working memory. It isn’t entirely clear what separates the two from the introduction.

“High emotion reactivity could increase the challenge of disengaging from salient negative content in WM could provide a mechanistic explanation of trait rumination”. I’m not sure I fully understand this sentence.

It would be appropriate to report how many outliers were detected and winsorized.

“we observed small nonsignificant associations”. I would strongly encourage reframing to be more oriented toward “we did not observe significant associations”. The original phrasing insinuates the potential presence of an effect, but this was not observed.

I would be inclined to suggest removing section 4.1 (“The relationship between rumination and emotion reactivity”), as this is not the central focus of the primary questions and hypotheses driving this paper. This might allow a more streamlined focus on the working memory components.

Why were positive image stimuli not included in analyses? I understand hypotheses were focused on negative stimuli, but it seems unnecessary to completely exclude this data.

Reviewer #2: In this study, the authors investigate how emotions can impact cognition, specifically examining hot and cold working memory in association with rumination and emotional reactivity. The study addresses an interesting topic. However, I have several concerns regarding the manuscript and their methodology, and specifically the analytical approach in its current form. My recommendations are listed below:

Major Comments:

1. The introduction needs to be rewritten to provide clarity regarding operationalization of the constructs and explanation of theories.

a. The authors list so many different constructs in the introduction, which gives the indication that they are all going to be variables explored in the results. For example, emotion dysregulation and executive function were mentioned in the introduction, but neither of these are measure of interest. It is unclear how they relate to the paper overall.

b. On page 3, the authors indicate “three key emotion processes: emotion reactivity, emotion dysregulation, and rumination . . .”. However, few lines later they define emotion regulation instead. Emotion regulation and emotion dysregulation are different constructs and the authors need to specify which one they are referring to. It seems that emotion dysregulation was not directly measured and majority of the text in this manuscript is discussing emotion regulation rather than emotion dysregulation.

2. The design, materials and methods section needs a major revision and additional clarification.

a. Under participants and procedures, the authors mention “difficulties with Urgency.” This is out of nowhere - what are they referring to as urgency here? More information is needed.

b. On page 7, the authors indicate, “After completing an online assessment of self-report measures, individuals who passed attention check were invited to the laboratory for a 2.5-hour session …” however, there is no indication of what the self-report measure or attention task was. This information must be provided.

c. For the measure, the authors must provide psychometric properties (i.e., internal consistency, Cronbach’s alpha) calculated from their current sample. Currently they only provide internal consistency reported by other studies.

d. The author refers to the Memory Affective Flexibility Task as novel, but there is no explanation of what about the task is novel.

i. The task figure needs to be updated - it is blurry and very difficult to follow. Also, the figure needs a more descriptive caption.

e. On page 9, the authors indicate “Allen et al., found no evidence for a speed-accuracy tradeoff.” I understand that refers to the study that looked at the same dataset, but to help the reader they should provide more details here, such as how the tradeoff was calculated. The reader should not be expected to go back and forth between papers.

f. On page 10, the authors stated “The limited associations suggest that RT may reflect individual differences in overall processing speed or response style rather than emotional valence specific effect, as suggested in the preliminary validation of MFT.” Where is this validation?

i. They further talk about “switching scores,” but thus far in the paper there is no explanation of what that score is or how it is measured.

g. What were the statistical assumptions that they tested?

h. The authors had to pivot from their pre-registered analysis that indicates that all of their analysis here are considered exploratory, and they need to explicitly state that.

3. The analytical approach and results are concerning and need to be reconsidered.

a. Fit indices (TLI < -4, CFI ~ 0, RMSEA = 0.63, SRMR = 0.15) indicates a serious issue where the model seems to be mis-specified. Given this serious misfit the SEM models should not be interpreted. A better or more interpretable approach, such as simple regression, would be a more appropriate option.

b. The authors report SRMR threshold of > 0.06 - could this be a typo? Did they mean SRMR threshold of < 0.06?

c. The combined use of winsorization and FIML can bias the estimates and standard errors. Winsorization changes the distribution and variance structure, whereas FIML assumes the data is raw data. The authors should provide a justification for using both of these approaches.

4. The discussion is very difficult to understand in terms of the structure and information that were provided in the discussion. Therefore, I recommend organizing it around the research questions/hypotheses and systematically comparing the findings with previous research. The study only had two hypotheses presented, but the discussion is discussing many more points than those relevant to the hypotheses.

Minor comment

• Need to consistently bold headings

• Line 71 should use parentheses instead of commas for clarity

Reviewer #3: This manuscript investigates how rumination and emotional reactivity relate to the processing of affective (“hot”) and neutral (“cold”) content in working memory. The authors use a modified n-back paradigm they developed in a previously published paper (Allen et al., 2025 Front. Psychiatry). The paradigm is a 3 (n: 1, 2, 3) x 3 (Valence: Negative, Positive, Neutral) design. On a given trial, participants are instructed to recall if they had previously seen an image at the n-level (WM updating) or to rate the image valence (affective flexibility). The main analytic approach is to use structural equation modeling (SEM) to test their predictions. Applying SEM to accuracy and RT (separately) produced poor fits and the only significant correlation of interest was between Rumination and Emotional Reactivity. However, the authors find no significant link between WM and rumination or emotional reactivity. The authors conclude by suggesting the correlation between rumination and emotional reactivity supports existing research, and that their null results highlight the challenge to study affect and cognition.

I appreciate the transparency of the manuscript and the fact that the hypotheses and analysis plan were preregistered, even though the study produced null results. However, I believe there are several conceptual and methodological weaknesses that make the manuscript unsuitable for sharing in its current form.

Major Concerns

1. The biggest concern in this manuscript is the purported ceiling effects of WM accuracy under both valence conditions. These ceiling effects – which would need to be supported statistically -- would compromise the ability to effectively test the authors’ core hypotheses, which hinge on testing individual differences in WM performance using correlational designs and SEM. Key hypotheses hinge on accuracy, not just RTs, so this is a serious concern.

2. In line with the first point, even though the SEM approach was preregistered, this was not an appropriate statistical method to choose. The model fits are extremely poor (RMSEA > 0.60). Thus, the takeaway from the modeling is not “our hypotheses were not supported” but instead that this was the wrong analytical decision. Given the small variance and non-normality (reported in the Shapiro-Wilks tests), the authors could instead focus on regression analyses or mixed-effects modeling (with trial-level data)

3. The Results section is severely underreported, and there are no inferential statistics assessing the results from the MAFT. The authors state in the discussion (Page 19, Lines 365-367) that “…participants responded less accurately and more slowly during trials with negative stimuli…”. This claim relies solely on interpreting descriptive statistics. The authors should use inferential statistics (e.g., t-test, ANOVA) to provide direct evidence for this claim.

4. The details of the procedure of the MAFT are underspecified in the Methods. Specifically, the details of the affective flexibility (valence rating) are not well detailed. Do participants report the valence of the current image or the valence of image n in the n-back? Also, only data in relation to the traditional n-back is reported but there is no inclusion of responses related to the affective flexibility portion of the task. If this is not of interest, why not just use a normal n-back with valenced stimuli? And, in its current form of reporting, the title suggesting “insights from a novel experimental paradigm” is not warranted.

5. The use of “hot” and “cold” WM is idiosyncratic and not well established in the literature. It seems what the authors are essentially studying is how WM functions in the presence of affective vs. neutral information. The language could be changed to fit better in the literature.

Minor Concerns

a) At the beginning of the Results section (Page 20, Line 250), the authors state, “All participants attained > 50% omission error rates on MAFT n-back trials”. I think this may be a typo and they mean “< 50%” (less than), otherwise not responding on over half the trials is a big issue.

b) The full reporting of demographics in Table 1 is unnecessary and takes up valuable space.

**********

what does this mean?). If published, this will include your full peer review and any attached files.

If you choose “no”, your identity will remain anonymous but your review may still be made public.

**Do you want your identity to be public for this peer review?**  For information about this choice, including consent withdrawal, please see our Privacy Policy

Reviewer #1: **Yes:**  Peter Kirk

Reviewer #2: No

Reviewer #3: No

**********

[NOTE: If reviewer comments were submitted as an attachment file, they will be attached to this email and accessible via the submission site. Please log into your account, locate the manuscript record, and check for the action link "View Attachments". If this link does not appear, there are no attachment files.]

To ensure your figures meet our technical requirements, please review our figure guidelines: https://journals.plos.org/plosone/s/figures

You may also use PLOS’s free figure tool, NAAS, to help you prepare publication quality figures: https://journals.plos.org/plosone/s/figures#loc-tools-for-figure-preparation.

NAAS will assess whether your figures meet our technical requirements by comparing each figure against our figure specifications.

# Author comment: Author response to Decision Letter 1

**DOI:** 10.1371/journal.pone.0360749.r002

27 Jul 2026

Reply letter

Academic editor

1. Please ensure that your manuscript meets PLOS ONE's style requirements, including those for file naming.

Reply: The authors apologize for not adhering to these requirements previously this should now be amended.

2. Please include a complete copy of PLOS’ questionnaire on inclusivity in global research in your revised manuscript. Our policy for research in this area aims to improve transparency in the reporting of research performed outside of researchers’ own country or community. The policy applies to researchers who have travelled to a different country to conduct research, research with Indigenous populations or their lands, and research on cultural artefacts. The questionnaire can also be requested at the journal’s discretion for any other submissions, even if these conditions are not met. Please find more information on the policy and a link to download a blank copy of the questionnaire here: https://journals.plos.org/plosone/s/best-practices-in-research-reporting. Please upload a completed version of your questionnaire as Supporting Information when you resubmit your manuscript.

Reply: Apologies for not including this. See file “S2 Inclusivity-in-global-research-questionnaire-Effect of Affect” for PLOS’ questionnaire on inclusivity in global research in the resubmission.

3. Thank you for stating the following in the Acknowledgments Section of your manuscript: “This work was funded by a grant from the Peder Sather Foundation and the corresponding author submitted the manuscript while on a Fulbright scholarship to UC Berkeley. The University of Bergen funded the work through coverage of the article processing charges.”

We note that you have provided funding information that is not currently declared in your Funding Statement. However, funding information should not appear in the Acknowledgments section or other areas of your manuscript. We will only publish funding information present in the Funding Statement section of the online submission form.

Please remove any funding-related text from the manuscript and let us know how you would like to update your Funding Statement. Currently, your Funding Statement reads as follows:

“The Peder Saether Foundation provided support for participant payment and for two meetings of the authors. Corresponding author is a visiting researcher at UC Berkeley on a Fulbright scholarship at the time of submission. The University of Bergen funded the article processing charges.” Please include your amended statements within your cover letter; we will change the online submission form on your behalf.

Reply: Apologies for misplacing this. The statement has been updated more in line with the PLOS one guidelines: “The Peder Saether Foundation used for participant payment and for two meetings of the authors (Peder Sather Grant funding period: October 2022-September 2025 awarded to SJ and AH). EHR was a visiting researcher at UC Berkeley on a Fulbright scholarship at the time of submission funding period: October 2025 to April 2026). Open access funding was provided by the University of Bergen. Funders had no part in the design, analysis or preparation or decision to publish the current study.”

This text has been added to the revised cover letter and removed from the manuscript. Please feel free to change the online submission form on my behalf.

4. We note that the grant information you provided in the ‘Funding Information’ and ‘Financial Disclosure’ sections do not match. When you resubmit, please ensure that you provide the correct grant numbers for the awards you received for your study in the ‘Funding Information’ section.

Reply: Funding text is removed from the manuscript.

5. Thank you for stating the following financial disclosure: “The Peder Saether Foundation provided support for participant payment and for two meetings of the authors. Corresponding author is a visiting researcher at UC Berkeley on a Fulbright scholarship at the time of submission. The University of Bergen funded the article processing charges.” Please state what role the funders took in the study. If the funders had no role, please state: "The funders had no role in study design, data collection and analysis, decision to publish, or preparation of the manuscript." If this statement is not correct you must amend it as needed.Please include this amended Role of Funder statement in your cover letter; we will change the online submission form on your behalf.

Reply: This should also be covered by the points above.

6. When completing the data availability statement of the submission form, you indicated that you will make your data available on acceptance. We strongly recommend all authors decide on a data sharing plan before acceptance, as the process can be lengthy and hold up publication timelines. Please note that, though access restrictions are acceptable now, your entire data will need to be made freely accessible if your manuscript is accepted for publication. This policy applies to all data except where public deposition would breach compliance with the protocol approved by your research ethics board. If you are unable to adhere to our open data policy, please kindly revise your statement to explain your reasoning and we will seek the editor's input on an exemption. Please be assured that, once you have provided your new statement, the assessment of your exemption will not hold up the peer review process.

Reply: Data will be shared upon publication in OSF from osf.io/ravqy. This has now been added to the cover letter and manuscript (see line 478)

Data Availability:

The data underlying the results presented in the study are available from osf.io/ravqy.

7. Your ethics statement should only appear in the Methods section of your manuscript. If your ethics statement is written in any section besides the Methods, please move it to the Methods section and delete it from any other section. Please ensure that your ethics statement is included in your manuscript, as the ethics statement entered into the online submission form will not be published alongside your manuscript.

Reply: The ethics statement has been moved to the subheading “Design, materials and methods”, line 127-132. Ethical approval for the study was obtained from the Committee for Protection of Human Subjects at the University of California, Berkeley (CPHS protocol ID’s 2024-01-17038 & 2020-07-13459). The study was conducted in accordance with the local legislation and institutional requirement. Participants provided their written informed consent to participate in this study.

8. We note that Figure 1 in your submission contain copyrighted images. All PLOS content is published under the Creative Commons Attribution License (CC BY 4.0), which means that the manuscript, images, and Supporting Information files will be freely available online, and any third party is permitted to access, download, copy, distribute, and use these materials in any way, even commercially, with proper attribution. For more information, see our copyright guidelines: http://journals.plos.org/plosone/s/licenses-and-copyright. We require you to either (1) present written permission from the copyright holder to publish these figures specifically under the CC BY 4.0 license, or (2) remove the figures from your submission:

1. You may seek permission from the original copyright holder of Figure(s) [#] to publish the content specifically under the CC BY 4.0 license.

We recommend that you contact the original copyright holder with the Content Permission Form (http://journals.plos.org/plosone/s/file?id=7c09/content-permission-form.pdf) and the following text: “I request permission for the open-access journal PLOS ONE to publish XXX under the Creative Commons Attribution License (CCAL) CC BY 4.0 (http://creativecommons.org/licenses/by/4.0/). Please be aware that this license allows unrestricted use and distribution, even commercially, by third parties. Please reply and provide explicit written permission to publish XXX under a CC BY license and complete the attached form.”

Please upload the completed Content Permission Form or other proof of granted permissions as an "Other" file with your submission. In the figure caption of the copyrighted figure, please include the following text: “Reprinted from [ref] under a CC BY license, with permission from [name of publisher], original copyright [original copyright year].”

2. If you are unable to obtain permission from the original copyright holder to publish these figures under the CC BY 4.0 license or if the copyright holder’s requirements are incompatible with the CC BY 4.0 license, please either i) remove the figure or ii) supply a replacement figure that complies with the CC BY 4.0 license. Please check copyright information on all replacement figures and update the figure caption with source information. If applicable, please specify in the figure caption text when a figure is similar but not identical to the original image and is therefore for illustrative purposes only.

Reply: Figure 1 has now been replaced with a figure complying with 2. And is cited according to CC BY 4.0 requirements in the current manuscript (See figure 1 caption).

Reviewer 1

Major

• While commendable for preregistering the study, I cannot seem to access the preregistration document. A publicly-accessible link is necessary in order to critically evaluate adherence to preregistered analyses.

Reply: Shareable link added below subheading “Design, materials and methods”, line 133-134.

• It’s not entirely clear to me why an SEM approach was adopted. I think the justification could be made a lot clearer. Additional, non-preregistered exploration of relationships within a regression or partial spearman rank framework could be insightful. Moreover, was age or any other demographic variables included as covariates?

Reply: We have taken the reviewers comments into consideration and made major changes to our statistical approach. See reply to comment from REV 3, major concerns, point 1 and 2.

• Many of the measures and analyses might benefit from more granularity. The stimuli, subjective measures, and behavioral measures are quite broadly categorized and averaged. For example, the stimuli have been split into negative vs neutral stimuli. However, the impact of threat-relevant stimuli (inducing fear/anxiety) will likely have very different impacts on cognition to sad or disgusting stimuli. Summary statistics were also used for questionnaires and n-back data. Might other ways of processing such data give more detailed insight? For example, d prime or drift diffuson modeling of n-back data? Focusing on questionnaire items which showed relatively larger variability and less floor effects? I am not suggesting conducting a myriad of tests or any form of data fishing. Rather, considering a select number of supplementary, non pre-registered analyses that the researchers think could be useful. Such analyses could either: 1) reveal preliminary evidence for more nuanced relationships otherwise obscured by more “simple” analyses, with any inferences described as tentative; or 2) support the notion that these represent true null effects, no matter which way the data is analyzed.

Reply: We thank the reviewer for these helpful suggestions. We are not equipped to comment on the set of specific-emotions evoked by the IAPS images presented in the MAFT, as the standard ratings for these images are reported on arousal and valence dimensions. However, we were able to determine that the “negative” images in the MAFT set were selected for a combination of high arousal and negative valence. Whereas the “neutral” images were selected for a combination of low arousal and intermediate (i.e., neither positive nor negative) valence. We have added this description along with means and standard deviations of the two images categories to the method section (line 200-206).

We have also clarified in the text throughout that this study investigated affect, and not emotions, but emotion processes. The following definition have been added on line 50-53:

“Emotion processes are dependent on affective states, defined as pleasant or unpleasant feelings of valence and a level and arousal, which is considered continuously involved in cognition, including problem-solving and decision-making [Wilson-Mendenhall et al., 2013].”

To address concerns regarding the level of granularity in the analyses, additional exploratory analyses were conducted using both the ERS subscales (Sensitivity, Persistence, and Arousal/Intensity) and the different n-back conditions. (See line 266-271). Due to this substantial shared variance, including the subscales as separate predictors in subsequent analyses would provide limited additional information and could introduce redundancy in the models. Therefore, the ERS total score was retained as the primary measure of emotional reactivity in further analyses. The 2-back and 3-back conditions were examined to assess whether increasing cognitive load in the 3-back condition added meaningful variance beyond 2-back. Although some statistical differences were observed, the overall pattern of results was highly similar across conditions. This approach was considered theoretically appropriate, while also reducing model complexity and redundancy in subsequent analyses. A composite score averaging performance across the 2-back and 3-back conditions was computed for subsequent analyses.

• The null results are largely discussed in the context of potential methodological limitations. This is important to keep in the discussion. However, it would be particularly beneficial if there was a paragraph or two commenting on what these results mean at face value. In other words, if these results represent a true null, what does this tell us about our theories of emotion and working memory? It is equally important to consider the possibility of null results being either true nulls or false negatives.

Reply: Thank you, we agree that this is an interesting topic that should be explored. As also requested by reviewer 2, the discussion has been re-organized and the introductory paragraphs of the discussion now address what our results could mean at face value if they are true nulls, by comparing it to recent investigations of the neural separability hypothesis (line 354-366).

Minor

It might be helpful to briefly introduce hot vs cold memory in the abstract.

Reply: The distinction between “hot” and “cold” working memory has now been clarified on line 24 in the abstract, reading as follows “In the present study, we investigated differences in negative (hot) and neutral (cold) working memory processing in relation to measures of rumination and emotion reactivity…”.

Be clear on the distinction between general executive functioning and working memory. It isn’t entirely clear what separates the two from the introduction.

Reply: The language is cleaned and specified so that the distinction between the two constructs is underscored. Changes are made on lines 63-70 of the introduction.

“High emotion reactivity could increase the challenge of disengaging from salient negative content in WM could provide a mechanistic explanation of trait rumination”. I’m not sure I fully understand this sentence.

Reply: Indeed, there was a missing word. We have added “which” to the sentence, and it now reads as follows: “High emotion reactivity could increase the challenge of disengaging from salient negative content in WM, which could provide a mechanistic explanation of trait rumination”, line 111-113.

It would be appropriate to report how many outliers were detected and winsorized.

Reply: We have added this for transparency “ In total, winsorization was applied to 10 cases, affecting accuracy variables (n2 neutral: n = 1; n3 neutral: n = 1; n2 negative: n = 1; n3 negative: n =1) and RT variables (n2 neutral: n = 2; n3 neutral: n = 1; n2 negative: n = 1; n3 negative: n = 2) in Supporting Information.

“we observed small nonsignificant associations”. I would strongly encourage reframing to be more oriented toward “we did not observe significant associations”. The original phrasing insinuates the potential presence of an effect, but this was not observed.

Reply: We have rephrased and made the language clearer according to the suggestion and the sentence now reads: “Contrary to predictions, we did not observe significant associations of either hot or cold WM indices with rumination or emotion reactivity”. line 351-353.

I would be inclined to suggest removing section 4.1 (“The relationship between rumination and emotion reactivity”), as this is not the central focus of the primary questions and hypotheses driving this paper. This might allow a more streamlined focus on the working memory components.

Reply: Section 4.1 (“The relationship between rumination and emotion reactivity” is now removed and our main findings, null results, have been discussed in a broader affective neuroscientific framework in two new paragraphs (line 354-379) that explores possible reasons for the lack of association between all three constructs (RRS, ERS, hot and cold WM).

Why were positive image stimuli not included in analyses? I understand hypotheses were focused on negative stimuli, but it seems unnecessary to completely exclude this data.

Reply: Thank you, we agree that this should be clarified. The focus on negative affective stimuli was adopted to investigate possible cognitive mechanisms related to challenges with emotion regulation, whereas positive affective stimuli or task-relevant stimuli has shown improved working memory capacity (Schweizer et al., 2019). Several previous studies that use the n-back paradigm have found that negative affective stimuli reduce working memory capacity, an effect that is supported by neuroimaging studies (for a review, see Huang et al., 2025). Furthermore, positive stimuli used in laboratory studies of the working memory are considered low in affective significance and are found to be less reliable than unpleasant stimuli (for a review, see Schweizer et al., 2019). Sections of this argumentation is now added in the introduction of the manuscript to clarify methodological choices, line 78-84

Reviewer 2

Major:

1. The introduction needs to be rewritten to provide clarity regarding operationalization of the constructs and explanation of theories.

a. The authors list so many different constructs in the introduction, which gives the indication that they are all going to be variables explored in the results. For example, emotion dysregulation and executive function were mentioned in the introduction, but neither of these are measure of interest. It is unclear how they relate to the paper overall.

Reply: Thank you. In the original draft we presented executive functions first before introducing working memory, because of their hierarchical relationship. We agree that this caused misleading assumptions of the focus of the article. To have clearer language and be more precise, we have changed the text in two places of the introduction. Firstly, line 63-84 has been refocused around the concept of Working Memory and not EFs , and then we have removed an overly confusing clause (“negative emotions can interfere with EF”). We have also clarified the distinction between emotion processes (emotion reactivity and regulation) and affect, and provided a definition of affect to underscore the limitations implicit in the use of these constructs, line 50-53.

b. On page 3, the authors indicate “three key emotion processes: emotion reactivity, emotion dysregulation, and rumination . . .”. However, few lines later they define emotion regulation instead. Emotion regulation and emotion dysregulation are different constructs and the authors need to specify which one they are referring to. It seems that emotion dysregulation was not directly measured and majority of the text in this manuscript is discussing emotion regulation rather than emotion dysregulation.

Reply: Thank you for pointing this out. It is true that we intend to refer to emotion regulation throughout this article and any mention of dysregulation is now removed.

2. The design, materials and methods section needs a major revision and additional clarification (major).

a. Under participants and procedures, the authors mention “difficulties with Urgency.” This is out of nowhere - what are they referring to as urgency here? More information is needed.

Reply: We have now clarified this by removing “difficulties with Urgency.” and instead given more detail by adding : “difficulties with Emotion-related impulsivity (ERI) (i.e characterized by impulsive cognition and behavior in response to heightened emotions [60], captured by Negative and Positive Urgency scales.”, line 140-142

b. On page 7, the authors indicate, “After completing an online assessment of self-report measures, individuals who passed attention check were invited to the laboratory for a 2.5-hour session …” however, there is no indication of what the self-report measure or attention task was. This information must be provided.

Reply: We have clarified this by adding “Measures were administered online, and they included 3 embedded items to capture poor attention (e.g., for data quality, please choose “1” for this item).”, line 148-150.

c. For the measure, the authors must provide psychometric properties (i.e., internal consistency, Cronbach’s alpha) calculated from their current sample. Currently they only provide internal consistency reported by other studies.

Reply: This is changed to the current sample, line 159-160

d. The author refers to the Memory Affective Flexibility Task as novel, but there is no explanation of what about the task is novel.

Reply: Thank you for pointing this out. Although the n-back paradigm is well known, the MAFT is, to our knowledge, the first affective n-back that includes both switching and n-back trials. However, as we have used only n-back trials, we agree that the use of novel was misleading. We have clarified our use of the MAFT under subheading 2.1.3, (lines 170-182) and removed the word "novel" in the title of the article.

e. The task figure needs to be updated - it is blurry and very difficult to follow. Also, the figure needs a more descriptive caption.

Reply:

f. On page 9, the authors indicate “Allen et al., found no evidence for a speed-accuracy tradeoff.” I understand that refers to the study that looked at the same dataset, but to help the reader they should provide more details here, such as how the tradeoff was calculated. The reader should not be expected to go back and forth between papers.

Reply: We added “in bivariate correlations shown as negative correlations” and “ (all r´s > 0.65)” on line 227, as well as “and trial types, and suggested that RT might reflect speed of processing rather than functioning as a WM-specific index.” to help the reader.

g. On page 10, the authors stated “The limited associations suggest that RT may reflect individual differences in overall processing speed or response style rather than emotional valence specific effect, as suggested in the preliminary validation of MFT.” Where is this validation?

Reply: To better show to the validation we added the clarification stated above (f.).

h. They further talk about “switching scores,” but thus far in the paper there is no explanation of what that score is or how it is measured.

Reply: We agree this might be unclear, so we added the following: “Although the task was developed to assess WM and switching (cognitive flexibility), recent work casts doubt on the validity of the switching indices. Accordingly, we focus on WM scores only here.” on line 179-182.

i. What were the statistical assumptions that they tested?

Reply: We have made this more clear in our method section under 2.2 Data analysis

The authors had to pivot from their pre-registered analysis that indicates that all of their analysis here are considered exploratory, and they need to explicitly state that.

Reply: We have added a transparency section to clarify this on 2.3.1 Deviations from the preregistration.

3. The analytical approach and results are concerning and need to be reconsidered (major).

a. Fit indices (TLI < -4, CFI ~ 0, RMSEA = 0.63, SRMR = 0.15) indicates a serious issue where the model seems to be mis-specified. Given this serious misfit the SEM models should not be interpreted. A better or more interpretable approach, such as simple regression, would be a more appropriate option.

Reply: We have made major changes to our statistical approach. See reply to comment from REV 3, major concerns, point 1 and 2. We have added a transparency section to clarify this on 2.2.1 Deviations from the preregistration.

b. The authors report SRMR threshold of > 0.06 - could this be a typo? Did they mean SRMR threshold of < 0.06?

Reply: Yes, this is a typo and has been corrected to < 0.06.

c. The combined use of winsorization and FIML can bias the estimates and standard errors. Winsorization changes the distribution and variance structure, whereas FIML assumes the data is raw data. The authors should provide a justification for using both of these approaches.

Reply: Thank you for raising this methodological issue. As the primary analysis has been revised from the pre-registered SEM analysis to multiple linear regression, FIML is no longer employed in the current analyses. This is clarified in our section 2.3.1 Deviations from the preregistration.

4. The discussion is very difficult to understand in terms of the structure and information that were provided in the discussion. Therefore, I recommend organizing it around the research questions/hypotheses and systematically comparing the findings with previous research. The study only had two hypotheses presented, but the discussion is discussing many more points than those relevant to the hypotheses.

Thank you. The three first paragraphs of the discussion are now organized around our two main hypotheses and are compared to previous findings. As requested by reviewer 1, these sections also address what our results could mean at face value if they are true nulls, by comparing it to recent investigations of the neural separability hypothesis. Section 4.1 has been removed and the rest of the discussion has been re-organized to increase clarity and headings are removed to increase flow. Taken together, we hope that the discussion is more coherent and takes necessary points for discussion into account.

Minor comment

Need to consistently bold headings

Reply: We have corrected this, as we also see that it was not consistent throughout the paper.

Line 71 should use parentheses instead of commas for clarity

Reply: This is corrected now.

Reviewer 3

Major concerns

1. The biggest concern in this manuscript is the purported ceiling effects of WM accuracy under both valence conditions. These ceiling effects – which would need to be supported statistically -- would compromise the ability to effectively test the authors’ core hypotheses, which hinge on testing individual differences in WM performance using correlational designs and SEM. Key hypotheses hinge on accuracy, not just RTs, so this is a serious concern.

Reply: Thank you for pointing this out. Shapiro-Wilk tests confirmed significant non-normality in accuracy variables (all ps < .001), consistent with ceiling effects in task performance. Log10 transformation was explored but did not resolve the non-normality (all ps < .001 post-transformation). This supports the interpretation that these reflect genuine ceiling effects rather than statistical artefacts. The 1-back condition was excluded from analyses as it places minimal demands on WM and would exacerbate these ceiling effects. RT was retained as an additional measure of WM performance given its sensitivity to cognitive load even under ceiling conditions in accuracy.

2. In line with the first point, even though the SEM approach was preregistered, this was not an appropriate statistical method to choose. The model fits are extremely poor (RMSEA > 0.60). Thus, the takeaway from the modeling is not “our hypotheses were not supported” but instead that this was the wrong analytical decision. Given the small variance and non-normality (reported in the Shapiro-Wilks tests), the authors could instead focus on regression analyses or mixed-effects modeling (with trial-level data).

Reply: We agree that this is an important methodological concern. Regarding the SEM misfit, we fully concur with the reviewers that the fit indices indicate severe model misspecification, rendering the SEM results uninterpretable, and have been moved to Supporting Information for transparency and are no longer interpreted as primary findings. In response to the concerns, multiple linear regression was chosen as an analytical approach. Regression models were conducted with rumination as the outcome, examining hot and cold accuracy, hot and cold RT, alongside emotional reactivity as predictors. The relevant changes are reflected in Section 2.2.1 of the revised manuscript.

3. The Results section is severely underreported, and there are no inferential statistics assessing the results from the MAFT. The authors state in the discussion (Page 19, Lines 365-367) that “…participants responded less accurately and more slowly during trials with negative stimuli…”. This claim relies solely on interpreting descriptive statistics. The authors should use inferential statistics (e.g., t-test, ANOVA) to provide direct evidence for this claim.

Reply: We have adopted a new statistical approach. On the issue of reporting on the MAFT, we refer to the published article by Allen and colleagues and added Allen et al. [59] found no evidence for a speed-accuracy tradeoff in bivariate correlations shown as negative correlations.. (line 222-223).

4. The details of the procedure of the MAFT are underspecified in the Methods. Specifically, the details of the affective flexibility (valence rating) are not well detailed. Do participants report the valence of the current image or the valence of image n in the n-back? Also, only data in relation to the traditional n-back is reported but there is no inclusion of responses related to the affective flexibility portion of the task. If this is not of interest, why not just use a normal n-back with valenced stimuli? And, in its current form of reporting, the title suggesting “insights from a novel experimental paradigm” is not warranted.

Reply: We now provide additional information regarding the design of the “n-back” (i.e., working memory) and “switch” (i.e., cognitive flexibility) trials (pg 9). In this study, we use the dataset described in the original publication of the MAFT (Allen et al., 2025); however, we examine emotion reactivity and rumination in relation to WM for negative and neutral visual stimuli (pg 7-8). We have now added a clarification regarding the exclusion of the “switch” trials from the present analyses on the basis of our hypotheses (pg 9). We agree that the use of the word “novel” is not warranted, and we have removed it from the title and the method section.

5. The use of “hot” and “cold” WM is idiosyncratic and not well established in the literature. It seems what the authors are essentially studying is how WM functions in the presence of affective vs. neutral information. The language could be changed to fit better in the literature.

Reply: We agree that the terms are quite specific but would like to keep the vocabulary for the following reasons: (1) “Hot” and “Cold” are operationalized terms of affective and neutral stimuli which indicate an experimental task. (2) Our research group has used this term in previous papers (see Allen et al.,[57]), and we would like to maintain similarity to reduce confusion of vocabulary for interested readers. (3) Recent prominent reviews use these terms, see examples added below:

Salehinejad, M. A., Ghanavati, E., Rashid, M. H. A., & Nitsche, M. A. (2021). Hot and cold executive functions in the brain: A prefrontal-cingular network. Brain and neuroscience advances, 5, 23982128211007769.

Mecha, P., Rodriguez‐Morales, M., & Sanchez‐Lopez, A. (2024). Components of hot and cold executive functions and their relations to different forms of stress resilience: A systematic review. Stress and Health, 40(5), e3439.

However, as reviewer 1 has requested the definitions of “hot” and “cold” in the abstract, this is now added to increase clarity. The distinction between “hot” and “cold” working memory can be found on line 24 of the abstract, reading as follows “we investigated differences in affective (hot) and neutral (cold) information processing in working memory…”.

Minor Concerns

a) At the beginning of the Results section (Page 20, Line 250), the authors state, “All participants attained > 50% omission error rates on MAFT n-back trials”. I think this may be a typo and they mean “< 50%” (less than), otherwise not responding on over half the trials is a big issue.

Reply: Yes, this is a typo and is now corrected.

b) The full reporting of demographics in Table 1 is unnecessary and takes up valuable space.

Reply: Table 1 is reduced to only include gender, age and education, with race/ethnicity removed in the table. A sentence describing race/ethnicity is added in the table description.

The authors thank the reviewers and editor for their time and expertise. The suggested revisions have substantially improved the validity of the findings and the presentation of the study. We now believe that the manuscript has improved considerably, and welcome any new comments or suggestions from the reviewers.

Attachment Submitted filename: ResponseLetter.docx

# Peer review: Decision Letter 1

**DOI:** 10.1371/journal.pone.0360749.r003

**Authors:** Desmond J. Oathes

30 Sep 2026

<p>The effects of affect: Working memory, rumination and emotion reactivity - Insights from an affective n-back task

PONE-D-25-66318R1

Dear Dr. Ronold,

We’re pleased to inform you that your manuscript has been judged scientifically suitable for publication and will be formally accepted for publication once it meets all outstanding technical requirements.

Within one week, you’ll receive an e-mail detailing the required amendments. When these have been addressed, you’ll receive a formal acceptance letter and your manuscript will be scheduled for publication.

An invoice will be generated when your article is formally accepted. Please note, if your institution has a publishing partnership with PLOS and your article meets the relevant criteria, all or part of your publication costs will be covered. Please make sure your user information is up-to-date by logging into Editorial Manager at Editorial Manager® and clicking the ‘Update My Information' link at the top of the page. For questions related to billing, please contact billing support.

If your institution or institutions have a press office, please notify them about your upcoming paper to help maximize its impact. If they’ll be preparing press materials, please inform our press team as soon as possible -- no later than 48 hours after receiving the formal acceptance. Your manuscript will remain under strict press embargo until 2 pm Eastern Time on the date of publication. For more information, please contact onepress@plos.org.

Kind regards,

Desmond J. Oathes

Academic Editor

PLOS One

Additional Editor Comments (optional):

Reviewers' comments:

Reviewer's Responses to Questions

**Comments to the Author**

Reviewer #1: All comments have been addressed

Reviewer #2: All comments have been addressed

**********

2. Is the manuscript technically sound, and do the data support the conclusions?

Reviewer #1: Yes

Reviewer #2: Yes

**********

3. Has the statistical analysis been performed appropriately and rigorously?

Reviewer #1: Yes

Reviewer #2: Yes

**********

4. Have the authors made all data underlying the findings in their manuscript fully available?

The PLOS Data policy

Reviewer #1: Yes

Reviewer #2: Yes

**********

5. Is the manuscript presented in an intelligible fashion and written in standard English?

Reviewer #1: Yes

Reviewer #2: Yes

**********

Reviewer #1: The authors have mostly addressed all my concerns and I have no further comments.

Signed,

Peter Kirk

Reviewer #2: The authors did a great job addressing the requested revisions. They also did a great job redoing the analysis.

**********

what does this mean?). If published, this will include your full peer review and any attached files.

If you choose “no”, your identity will remain anonymous but your review may still be made public.

**Do you want your identity to be public for this peer review?**  For information about this choice, including consent withdrawal, please see our Privacy Policy

Reviewer #1: **Yes:**  Peter Kirk

Reviewer #2: No

**********

# Editor report: Acceptance letter

**DOI:** 10.1371/journal.pone.0360749.r004

**Authors:** Desmond J. Oathes

PONE-D-25-66318R1

PLOS One

Dear Dr. Ronold,

I'm pleased to inform you that your manuscript has been deemed suitable for publication in PLOS One. Congratulations! Your manuscript is now being handed over to our production team.

At this stage, our production department will prepare your paper for publication. This includes ensuring the following:

* All references, tables, and figures are properly cited

* All relevant supporting information is included in the manuscript submission,

* There are no issues that prevent the paper from being properly typeset

You will receive further instructions from the production team, including instructions on how to review your proof when it is ready. Please keep in mind that we are working through a large volume of accepted articles, so please give us a few days to review your paper and let you know the next and final steps.

Lastly, if your institution or institutions have a press office, please let them know about your upcoming paper now to help maximize its impact. If they'll be preparing press materials, please inform our press team within the next 48 hours. Your manuscript will remain under strict press embargo until 2 pm Eastern Time on the date of publication. For more information, please contact onepress@plos.org.

You will receive an invoice from PLOS for your publication fee after your manuscript has reached the completed accept phase. If you receive an email requesting payment before acceptance or for any other service, this may be a phishing scheme. Learn how to identify phishing emails and protect your accounts at https://explore.plos.org/phishing.

If we can help with anything else, please email us at customercare@plos.org.

Thank you for submitting your work to PLOS One and supporting open access.

Kind regards,

PLOS One Editorial Office Staff

on behalf of

Dr. Desmond J. Oathes

Academic Editor

PLOS One
